# On the limited consensus of mountain pine beetle impacts on wildfire

**DOI:** 10.1007/s10980-023-01720-z

**Published:** 2023-07-13

**Authors:** D. C. Romualdi, S. L. Wilkinson, P. M. A. James

**Affiliations:** Institute of Forestry and Conservation, Daniels Faculty of Landscape, Architecture and Design, University of Toronto, 33 Willcocks St, Toronto, ON M5S 3B3 Canada

**Keywords:** Disturbance interaction, Forest insect pest, Forest health, Fire severity, Fire intensity

## Abstract

**Context:**

The mountain pine beetle (MPB; *Dendroctonus ponderosae*) is a native bark beetle whose outbreaks leads to widespread conifer forest mortality. Of particular concern to forest and wildfire managers is the influence of MPB outbreaks on wildfire via spatial legacies left in impacted forest stands. There is, however, limited consensus in the literature regarding how MPB outbreaks affect wildfire across western North America.

**Objectives:**

This meta-analysis aims to (1) summarize available evidence regarding MPB-wildfire interactions, and (2) identify environmental and methodological indicators associated with various wildfire responses (i.e., amplified, neutral, or dampened) post-outbreak.

**Methods:**

We include peer-reviewed publications focusing on MPB outbreaks and subsequent wildfire activity in forests across western Canada and the USA between 2000 and 2021. A classification scheme was used to examine attributes of each publication to assess which indicators contribute most to their associated wildfire response.

**Results:**

We found that spatial scale, forest fuels, and weather are main drivers of variation in wildfire response post-outbreak. Metrics of forest fuels and inclusion of weather data on a stand-scale are related to amplified fire responses, whereas dampened responses correspond to landscape-scale analyses. Furthermore, red-stage stands are associated with amplified fire response, whereas other stages are associated with dampened response—supporting current conceptual models of the importance of outbreak stage on wildfire.

**Conclusions:**

Advancing our understanding regarding drivers of wildfire responses post-MPB outbreak is key to developing accurate, and comparative research studies. These findings provide crucial information for wildfire, and forest management agencies, especially in forests newly exposed to this disturbance interaction under climate change.

**Supplementary Information:**

The online version contains supplementary material available at 10.1007/s10980-023-01720-z.

## Introduction

Forest landscapes are shaped by multiple and often interacting disturbances (Canelles et al. 2021) that have effects on landscape dynamics and ecosystem processes (Turner 2010). Natural disturbances impact forest landscape dynamics through the generation of spatial heterogeneity at multi-scales, as well as subsequent ecosystem functions and processes through long-term spatial legacies and succession (Turner 2010). These disturbances include abiotic agents such as drought, windstorms, landslides, and wildfires, as well as biotic agents such as insect outbreaks and disease (Turner 2005, 2010; Seidl et al. 2017). Further, forest harvesting is a large-scale anthropogenic disturbance that often interacts with natural disturbances (Canelles et al. 2021). The characteristics of natural disturbances (i.e., spatial extent, frequency, intensity, and severity) are influenced by geography, vegetation structure and composition, weather, and climate (Dale et al. 2001; Flannigan et al. 2009), resulting in a range of potential ecological outcomes for a single disturbance type (Seidl et al. 2017). The spatial legacies that disturbances leave behind in the form of residual forest structure, altered ecological function, and successional vegetation trajectories can affect future disturbances well after the original disturbance event has passed (James et al. 2011; Silins et al. 2014). These spatial legacies can interact with other disturbances to amplify or attenuate future disturbance behavior and associated outcomes (Turner 2010; Buma 2015; Seidl et al. 2017). However, there remain significant gaps in our understanding regarding how forest disturbances influence one another through space and through time, including their interactions with climate change induced drought via increasing temperatures (Seidl et al. 2017).

The most extensive natural disturbances in North American forests are outbreaks of forest insects and wildfire, each affecting millions of hectares annually (Natural Resources Canada 2020; Hoover & Hanson 2021; Fettig et al. 2022; National Forestry Database 2022). The mountain pine beetle (MPB—*Dendroctonus ponderosae* Hopkins) is a phloem feeding insect whose outbreaks result in widespread mortality of host trees (Safranyik and Carroll 2006) and create complex spatial patterns of forest mortality at the landscape scale. Outbreaks and consequent mortality alter landscape- and stand-scale forest fuel structure over time (Page and Jenkins 2007; Simard et al. 2011; Schoennagel et al. 2012; Harvey et al. 2014a). The influence of MPB outbreaks on forest structure and wildfire is one of the most well-studied disturbance interactions (Canelles et al. 2021; Fettig et al. 2021). These landscape-scale outbreaks have been observed across western Canada and the USA since the early 1900s (Safranyik and Carroll 2006; Taylor et al. 2006; Raffa et al. 2008) and research into the interactions between wildfire and outbreaks has mainly occurred since the 1990s (Jenkins 1990).

Changes in impacted stand structures has led to the characterization of four time-since-beetle (TSB) stages including: (1) green-stage (0–1; unattacked to year of attack); (2) red-stage (1–2 years post-outbreak); (3) gray-stage (3–10 years post outbreak); and (4) old-stage (10 + years post outbreak) (Simard et al. 2011). Each TSB stage is associated with distinct alterations to fuel size, loading, and connectivity (Simard et al. 2011). As MPB reach epidemic levels, stands transition from the green- to red-stage and in-stand foliar moisture content (%) levels significantly decrease (Jolly et al. 2012; Page et al. 2012; Jenkins et al. 2014). Red-stage stands then experience an accumulation of fine (1–10 h) fuels—such as needles and twigs—in the canopy and on the forest floor, therefore increasing needle litter and duff depth, coupled with a decrease in canopy fuels as needles fall off dying trees (Jenkins et al. 2008; Klutsch et al. 2009; Simard et al. 2011). Post-outbreak stands are characterized by the gray-stage which show an increase in coarse (100-h) fuel accumulation on the forest floor, including relatively large woody debris such as sticks and small branches, as well as a near complete absence of fine (live and dead) aerial fuels (Safranyik and Carroll 2006; Simard et al. 2011). Lastly, old-stage stands exhibit the highest amount of 1000-h woody fuels including whole host trees that have fallen to the ground (Simard et al. 2011).

MPB outbreaks also lead to notable changes in attacked stand fuel properties such that foliar moisture content, as well as the proportions of starch, sugar, and crude fat are found to substantially decrease, whereas the proportions of lignin, cellulose, and hemicellulose tend to increase with time since an outbreak (Jenkins et al. 2012, 2014; Jolly et al. 2012; Page et al. 2012). Such changes to MPB attacked fuel properties influence subsequent fire ignitability (i.e., time to ignition), combustibility (i.e., maximum fire temperature), consumability (i.e., biomass consumption), and sustainability (i.e., duration of flaming) (Page et al. 2012).

Despite a thorough conceptual understanding of MPB outbreaks, there are many sources of variability and uncertainty in the realized effects of MPB outbreaks on forest stand and fuel structure over time. Although outbreaks have occurred most extensively in lodgepole pine (*Pinus contorta*), ponderosa pine (*Pinus ponderosa*), and western white pine (*Pinus monticola*) stands throughout the interior of British Columbia, Canada, and across Rocky Mountain states in the USA (Meddens et al. 2012), other pine species are also susceptible. These species include the native eastern white pine (*Pinus strobus*) and jack pine (*Pinus banksiana*), as well as the non-native Scots pine (*Pinus sylvestris*) (Safranyik and Carroll 2006). Different species occur in different ecoregions, elevations, and have different pre-disturbance stand structures. MPB outbreaks also vary in terms of severity based on the pre-disturbance forest landscape and stand structure including forest composition and spatial configuration, as well as weather, and climate (Taylor et al. 2006). In combination, these factors determine the effects of MPB outbreak on forest stand and fuel structure over time, and weather, climate, and anthropogenic factors (e.g., ignitions) further influence the interaction with wildfire.

The effects of MPB on wildfire are also directly and indirectly affected by climate change. Associated increases in wildfire activity in western North America (Flannigan et al. 2009), changes in fire severity (Parks and Abatzoglou 2020), as well as observations of extreme or novel fire behavior in MPB-affected stands (Moriarty et al. 2019) has prompted a need for clarity regarding how wildfire is affected by MPB outbreaks. Rising winter temperatures are enabling forest insect pests to expand their ranges, exposing greater areas (Bentz and Klepzig 2014; Bentz et al. 2014; Cooke and Carroll 2017) and new ecosystems (Bentz et al. 2011). This has fueled larger and more widespread MPB-outbreaks in recent years including northward expansion into the Yukon, and eastward expansion into Alberta, Canada (Cooke and Carroll 2017). Climate change is promoting increases in area burned (Flannigan et al. 2005; Hanes et al. 2019), average fire size (Wang et al. 2020), and severe fire weather (Jain et al. 2022) leading to these disturbances becoming increasingly interconnected (Seidl et al. 2017). Concerns over forest health, as well as public safety (Schoennagel et al. 2017; Coogan et al. 2019), under enhanced wildfire activity, exposes a critical need to better understand wildfires’ interactions with MPB outbreaks and their spatial legacies to ensure effective management of fires (Sankey 2018).

At present, the effect of MPB outbreaks on wildfire remains mixed throughout the literature (Canelles et al. 2021; Fettig et al. 2022) and is insufficiently captured in fire and forest management strategies, as well as underlying policy (Sankey 2018; Leverkus et al. 2021). Although many studies have investigated the MPB-wildfire interaction, limited consensus remains among studies regarding its direction, magnitude, duration, and spatiotemporal variation. Some studies have idenitifed amplifying effects of MPB outbreaks on wildfire, including greater crown fire hazard (Ager et al. 2007; Perrakis et al. 2014), surface fire intensity (Page and Jenkins 2007; Hoffman et al. 2013), or both (Hoffman et al. 2013; Schoennagel et al. 2012). In contrast, other studies have identified dampening effects of outbreaks on wildfires (Crotteau et al. 2018). Reported dampened responses include reduced crown fire potential (Hoffman et al. 2015), reduced burn severity as captured by remotely sensed indices of ecological change (i.e., differenced normalized burn ratio − dNBR, and relative differenced normalized burn ratio − RdNBR) and/or changes to forest biomass and fuels post-fire (Meigs et al. 2016; Millar and Delany 2019). For example, a number of studies (Hoffman et al. 2015; Page and Jenkins 2007; Perrakis et al. 2014; Schoennagel et al. 2012) attribute amplified fire responses to alterations in fuel structure associated with relatively early TSB stages (i.e., red- and gray- stages) due to the observed accumulation of highly connected fine and coarse surface fuels (Hoffman et al. 2012). However, associated decreases in canopy fuel bulk density and foliar moisture with time since an outbreak has been found to influence wildfire metrics depending on variation in local environmental conditions (Hoffman et al. 2015; Millar and Delany 2019). Specifically, Hoffman et al. (2015) found that ambient wind speed controlled the direction of the impacts of MPB outbreak wherein rate of spread decreased as canopy biomass was reduced during early outbreak stages; yet, rate of spread increased under the influence of high and low wind velocities in a high mortality gray-stage stage stand. Likewise, other studies conclude neutral or non-significant impacts and attribute differences in wildfire metrics to the overriding effects of fire weather (Talucci and Krawchuk 2019; Hart and Preston 2020). For example, Hart and Preston (2020) argue that fire weather has a greater influence over daily area burned than changes in attacked stand fuel structure. Further, some studies report contrasting results within a single region or site depending on MPB outbreak stage or fire weather conditions (Klutsch et al. 2011; Harvey et al. 2014a; Hoffman et al. 2015; Agne et al. 2016; Sieg et al. 2017) highlighting the complexity of the MPB-wildfire interaction. In one of the more comprehensive studies of this subject, Harvey et al. (2014a), found that the influence of MPB outbreaks varied with time-since outbreak and fire weather.

Resolution of these contrasting results is necessary for the development of forest ecology best management practices, policy, and effective forecasting of future outbreaks. In an effort to resolve some of this uncertainty, we conduct a scoping review of MPB-fire interactions to investigate the drivers underlying the divergent results reported in the literature. We adopt a novel, ordination-based meta-analysis approach to investigate the drivers underlying the divergent results in the literature relating to wildfire response following MPB outbreaks across western Canada, and the USA. The objectives of our review are threefold: (1) summarize the available evidence regarding MPB-wildfire interactions, (2) identify environmental and methodological indicators associated with different disturbance interaction outcomes (i.e., amplified, neutral/non-significant, or dampened disturbance interactions), and (3) determine knowledge gaps and suggest future research priorities with the intent of facilitating the inclusion of MPB-wildfire interactions in relevant policy and management strategies. Previous reviews (Jenkins et al. 2014) have focused on summarizing findings from the literature, but none have done so with the explicit objective of determining why there is so little consensus among studies. We hypothesize that both environmental variables (e.g., fire weather, tree species, time-since-beetle outbreak) and methodological decisions (e.g., spatial scale, empirical vs modeled data, fire metric assessed) will influence disturbance interaction outcomes. Specifically, we hypothesize that an amplified fire response (i.e., an increase in fire severity or intensity) will be strongly associated with studies assessing red-stage stands conducted or modeled under low–moderate fire weather, while studies conducted under high–extreme fire weather across TSB-stages will conclude non-significant impacts of MPB outbreak due to the potentially overriding effect of fire weather on fire metrics. Further, we hypothesize that dampened fire (i.e., a decrease in fire severity or intensity) will be associated with studies assessing gray-or old-stage stands due to their reduced canopy fuel loads, or conducting landscape-scale analyses due to the lower spatial resolution of assessment. Lastly, we hypothesize that there will be an effect of geographic division (e.g., MPB affected provinces in western Canada, and states in western USA) and/or tree species on concluded MPB-wildfire interactions.

## Materials and methods

We undertook an extensive literature review by searching for peer-reviewed publications focusing on the role of MPB outbreaks on subsequent wildfire activity and associated outcomes (e.g., area burned) in host conifer forests across western Canada, and the USA. Using Web of Science, Google Scholar, University of Toronto Libraries, and Fire Research Institute databases, articles published between 2000 and 2021 were identified by selecting those with one or more of the following terms in the title, abstract, or keyword section: mountain pine beetle (*Dendroctonus ponderosae*; MPB); bark beetle; mountain pine beetle outbreak(s); wildfire/fire; fire metric; severity; intensity; spread rate; ignition; fuel; United States of America (USA); Canada; disturbance/disturbance interaction; and/or fire weather. While fire can influence future insect outbreaks (Fettig et al. 2021), here we focus on studies that examine the consequences of MPB outbreaks on wildfire. Using this approach, we generated a primary database of publications that measured one or more metrics of the following five attributes of wildfire following a MPB outbreak: (1) intensity; (2) severity; (3) ignition probability; (4) fire occurrence; and (5) area burned.

All wildfire attributes were not represented equally in our primary article database. Few studies examined the effects of MPB outbreaks on ignitions, occurrence, or area burned, whereas numerous studies examined severity and intensity (see Results). Consequently, we chose to further refine our study and to generate a secondary article database to specifically examine how fire *intensity* and *severity* are affected by MPB outbreaks via statistical analyses. Publications on fire-MPB interaction that were excluded from this analysis are summarized in Table S1. Emphasis on intensity and severity allowed us to better focus on the impacts of wildfire on forest structure and composition, forest health, and ecosystem services and functions. When classifying papers according to the fire metric examined, we used the definitions of Key and Benson (2006) regarding severity and intensity. However, intensity and severity were sometimes used interchangeably in some of the articles examined. Studies that measured or modeled ecological change in live forest vegetation, biomass structure, and soil characteristics post-fire were classified as a fire severity study, whereas those that measured or modeled the energy output of a fire during combustion were classified as a fire intensity study (Key and Benson 2006). Within both our primary and secondary database, each paper was classified using 14 categories of indicators. The categories included: fire metric, wildfire fuels, spatial scale, fire weather, fire weather category, TSB stage, outbreak intensity, MPB control plot, MPB range, tree species, ecoregion, decade of dataset, and geographic division. The response of wildfire to prior MPB outbreak concluded by the authors was also recorded. Spatial variation in MPB outbreak effects was not considered for classification as no studies reported on such variation within the fires examined. The secondary database used for statistical analyses include study site coordinates (if not directly stated in the article, coordinates were estimated using Google Maps) and associated ecoregion(s) based on the Level III Ecoregions of North America (United States Environmental Protection Agency 2022) to better visualize the distribution of data collection across Canada and the USA.

A common classification scheme was performed per publication wherein each of the 14 main categories of variables contained indicators to refine the attributes of each publication for a total of 48 indicators (Table 1). Indicator variables were recorded based on a binary presence (1) or absence (0) analysed within each study. This was done to enable analytical assessment of the indicators contributing most to the articles’ conclusion(s) regarding wildfire in response to MPB outbreaks. Specifically, studies were assigned a dampened, neutral, or amplified fire response based on whether the analyses and/or conclusions found that fire severity or intensity was decreased, unchanged, and/or increased, respectively, following a MPB outbreak. Publications that reported more than one wildfire response to MPB outbreak (e.g., an amplified and a neutral response associated with different MPB outbreak stages such as red-stage and gray-stage outbreaks, respectively) were duplicated in the database to ensure fire responses were associated with (and only with) the appropriate indicators according to each conclusion stated within the publication.

**Table 1:** Fourteen main categories and associated indicators with descriptors used to chart publications

Category	Indicators	Notes
Fire metric	Intensity measured, intensity modeled, severity measured, severity modeled	Each paper assigned one main fire metric of interest. Intensity/severity metric determined based on the definitions of Key & Benson 2006 (Key and Benson 2006)
Wildfire fuels	Fuels considered in analysis, and: Surface fuels, ladder fuels, canopy fuels, connectivity, drying rate/fuel size, fuel moisture	Presence (1) was awarded if fuel metrics were measured or modeled pre-fire Presence (1) subsequently awarded for individual measures of fuel metrics reported
Spatial scale	Individual tree/plot, stand, landscape	Presence (1) was awarded to multiple indicators if, for example, results were upscaled from plot to stand, or stand to landscape
Fire weather	Fire weather considered in analysis, and: temperature, relative humidity, precipitation, wind speed	Presence (1) was awarded if absolute values or fire weather indices were reported Presence (1) was subsequently awarded for individual aspects of fire weather variables reported or included in calculations for categorized burning conditions analysed within a study (e.g. Fire Weather Index (FWI) or remote automatic weather station (RAWS) data)
Fire weather category	Moderate, very high/extreme	Presence (1) was awarded if papers reported the fire weather relative to historical norms. Very high/extreme was awarded to papers using/reporting 90^th^ percentile weather variables or above
Time-since-beetle stage	Green, red, gray, old	Green; endemic, 0 years post-infestation, Red; 1–2 years, Gray; 3–10 years, Old; 10 + years
Outbreak intensity/severity	Outbreak intensity/severity considered, and: low, moderate, high	Presence (1) was awarded if a given outbreak intensity metric was measured or modeled Presence (1) subsequently awarded for the specific intensity level reported
MPB control plot	Includes MPB control plot	Presence (1) was awarded if papers compared results to a control plot that was unaffected by MPB
MPB range	MPB range included	Presence (1) was awarded if papers included a measure of MPB range
Tree species	Multiple species, and: Lodgepole pine (*Pinus contorta*); Ponderosa pine (*Pinus ponderosae*); Douglas-fir (*Pseudotsuga menziesii*); Western white pine (*Pinus monticola);* White bark pine (Pinus albicaulis); Pinyon pine (*Pinus monophylla*)	Presence (1) was awarded if papers included a measure of and/or comment on the dominant tree species in their study area/region
Ecoregion	Arizona/New Mexico Mountains; Blue Mountains; California Coastal Sage, Chaparral, and Oak Woodlands; Canadian Rockies; Cascades; Central Basin and Range; Chilcotin Ranges and Fraser Plateau; Columbia Mountains/Northern Rockies; Eastern Cascades Slopes and Foothills; Idaho Batholith; Klamath Mountains; Middle Rockies; North Cascades; Northwestern Great Plains; Sierra Nevada; Skeena-Omineca-Central Canadian Rocky Mountains; Southern and Baja California Pine-Oak Mountains; Southern Rockies; and Wasatch and Uinta Mountains	Information taken from publication directly or extracted based on site coordinates mapped in Google Earth. Ecoregions defined in https://www.epa.gov/eco-research/ecoregions-north-america
Decade of dataset	< 2000’s, 2000s, 2010s	Presence (1) was awarded to the associated decade of data collection per paper
Geographic division	Western Canada, western USA multiple, west NW (Idaho, Oregon, Washington), east NW (Montana, Wyoming), west SW (California), east SW (Arizona, Colorado, New Mexico, Utah)	Presence (1) was awarded to the geographic regions in which papers conducted their research
Fire response	Dampened, neutral, amplified	Dampened—limiting or antagonistic effect; neutral—no significant effect; amplified—enhancing or synergistic effect

Note that the Fuels considered indicator within the Wildfire fuels category was manually removed due to collinearity with other indicators following Hellinger transformation and hence not included in the PCA. Fire weather considered, fuel moisture, outbreak low, and outbreak moderate were removed using the findCorrelation function for the classification tree

### Statistical analyses

#### Unsupervised classification: principal components analysis (PCA)

To identify potential groupings of publications and associated fire conclusions among a set of indicator variables, we employed a PCA. All analyses were conducted in R Studio, version 4.2.1 (R Development Core Team 2022).To visualize and interpret the relative contributions of different indicators to variation in reported post-outbreak fire response, we used Principal Components Analysis (PCA) on all indicators in the following subset of categories: fire metric, wildfire fuels, spatial scale, fire weather, classified fire weather category, time since beetle (TSB) stage, outbreak intensity, MPB control plot, MPB range, tree species, decade of dataset, and geographic division (Table 1; note that Ecoregion was excluded from the PCA given that the number of studies associated with each ecoregion was very low). Data were Hellinger transformed prior to PCA to ensure the influence of double-zeros was avoided in the ordination (Zelený 2022). To retain as many indicators as possible, correlations between indicators were manually evaluated within each category using the Pearson correlation coefficient (Bourbonnais et al. 2014; Zuur et al. 2010) and indicators with a correlation coefficient greater than 0.8 were excluded from further analysis. This was done to ensure there were a sufficient amount of indicators left to proceed with further analysis (i.e., filtering indicators based on their significance to PC axes). A global PCA was conducted using the *prcomp* function (R Development Core Team 2022). Significant principal component (PC) axes were identified using the Broken-Stick criterion (Peres-Neto et al. 2003). A PC axis is considered significant when the proportion of variance explained by the ordination is greater than the proportion of variance expected under the Broken-Stick model.

The Broken-Stick criterion was also used to determine which indicator variables contribute significantly to each significant PC axis (Peres-Neto et al. 2003). In this way, we estimated the relative contribution of each indicator to variation in the response of fire to MPB. First, the loadings of each indicator variable were extracted for PC1 and PC2 using the *scores* function in **vegan** (Oksanen et al. 2022; R Development Core Team 2022), and subsequently squared and sorted from largest to smallest in value. Then, a simulated Broken-Stick distribution was generated using the *brokenStick* function in **PCDimension** (Coombes & Wang 2022) containing an equal number of components to indicators. An indicator variable was significantly related to a PC axis when the observed squared and ranked loading was greater than its associated rank’s loadings under the simulated Broken-Stick model. Significant ordination axes and indicators were plotted using the *ggbiplot* function (Wickham 2016). Points representing each study in these ordinations were color-coded to reflect the study’s reported impact of MPB on fire (i.e., dampened, neutral, or amplified).

#### Supervised classification: classification model

To determine which indicators lead to a given fire response in MPB-attacked stands, we used a multi-class classification tree analysis on all (nominal) responses in the secondary database. The *cor* function in the **corrgram** package (Wright 2021) was used to produce a correlation matrix of all associated indicators (R Development Core Team 2022), and we removed indicators with a Pearson’s correlation coefficient threshold of 0.8 using the *findCorrelation* function in the **caret** package (Kuhn 2022). This function assesses values of pairwise correlations and subsequently removes the variable in the pair which has a greater, average absolute correlation (R Development Core Team 2022). This resulted in the exclusion of fire weather considered, fuel moisture, outbreak low, and outbreak moderate when building the model. A single response column was created with dampened, neutral, and amplified variables. A multi-class classification tree model was generated using the *tree* function in the **tree** package (Ripley 2022). The tree package uses the response in a specified formula to conduct binary recursive partitioning and chooses splits from the variables on the right-hand side of the formula. These splits were based on deviance and are made to reduce impurity (Breiman et al. 1984; Ripley 1996, 2022). The resultant tree shows only indicators which were significant in classifying the fire responses.

## Results

### Available evidence regarding MPB–wildfire interactions

**Fig. 1:**
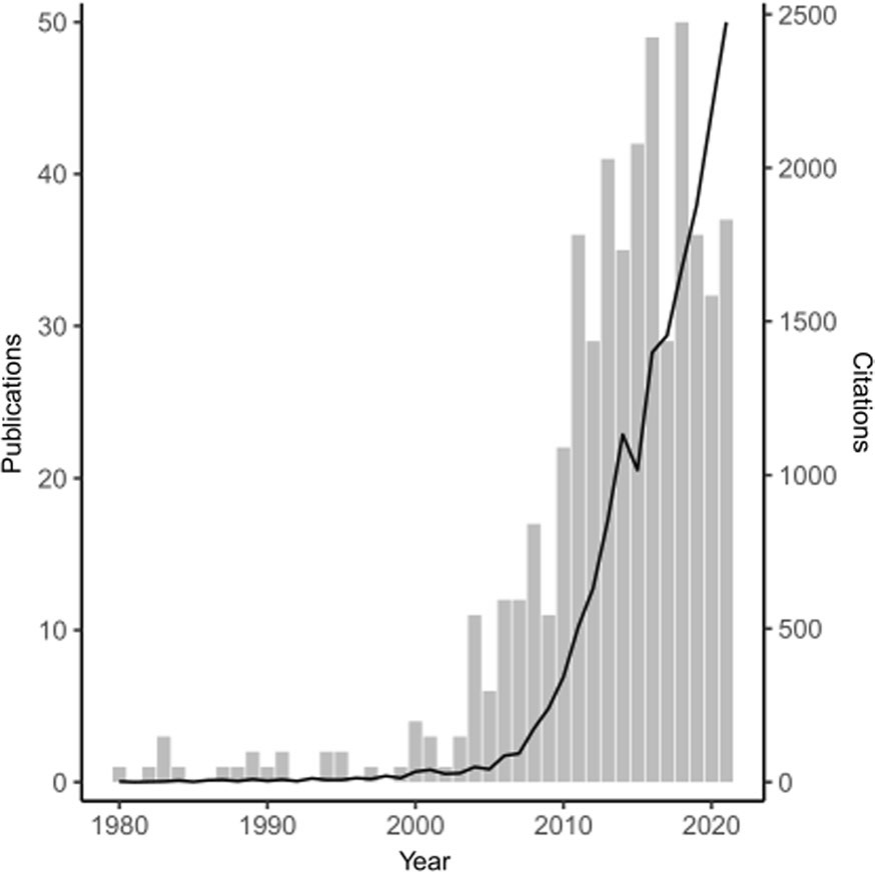
Number of publications (left y axis) and citations (right y axis) per year between 1980 and 2021, returned from Web of Science search of “Mountain pine beetle” and “*fire”. Gray bars represent the number of studies related to MPB and fire published per year, and the black line represents the number of citations on the topic of MPB and wildfire interactions per year

**Fig. 2:**
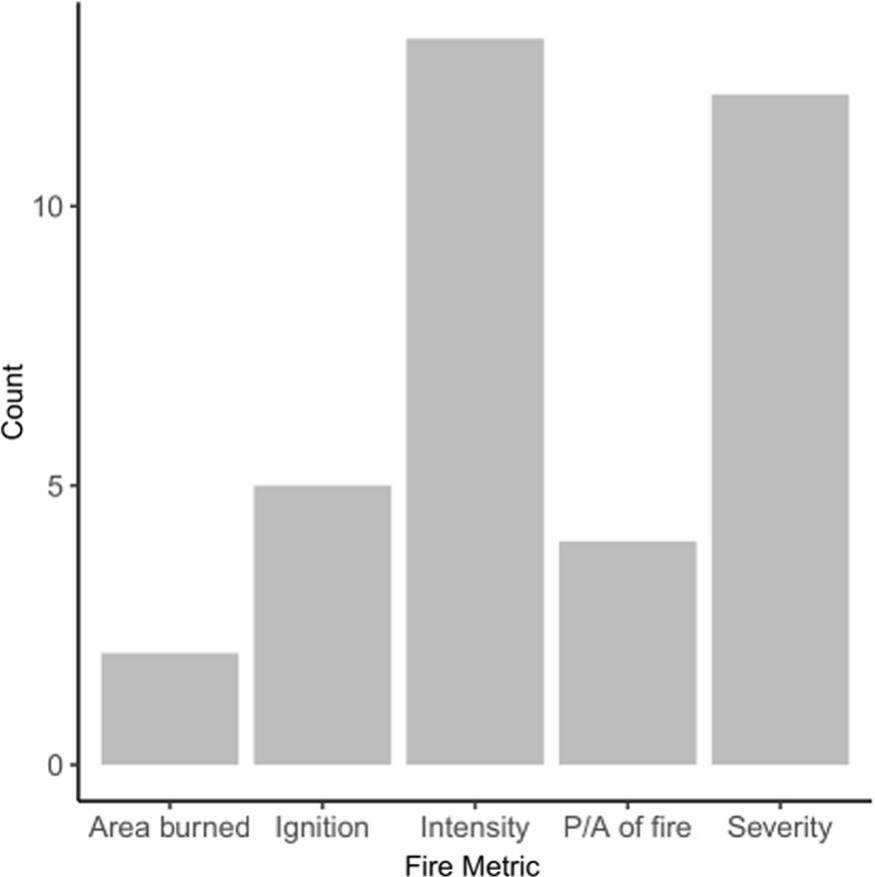
Grouping of the 27 papers in the primary database corresponding to the main fire behavior metric evaluated within each study. P/A represents ‘presence or absence’ of wildfire, and definitions proposed by Key and Benson (2006) were used to differentiate between fire intensity and fire severity. On the topic of wildfire activity following a MPB outbreak, we found most papers analyzed fire intensity and severity, followed by ignition, P/A of fire, and area burned, respectfully

Interest in how MPB outbreaks affect wildfire has risen sharply over the past three decades (Fig. 1). Although the number of publications appear to have peaked between 2016 and 2018, citations continue to rise (Fig. 1). In total we identified 27 pertinent peer-reviewed publications based on our specific search criteria. In brief, we selected papers that assessed the impact of MPB outbreak on subsequent fire response metrics in western Canada and the USA. Each paper was initially categorized according to the fire response metric examined. We found a range of different metrics examined, where categories included: intensity, severity, ignition probability, presence/absence of fire, and area burned. Most studies examined fire intensity and/or fire severity following an outbreak (*n* = 19/27; Fig. 2).

**Fig. 3:**
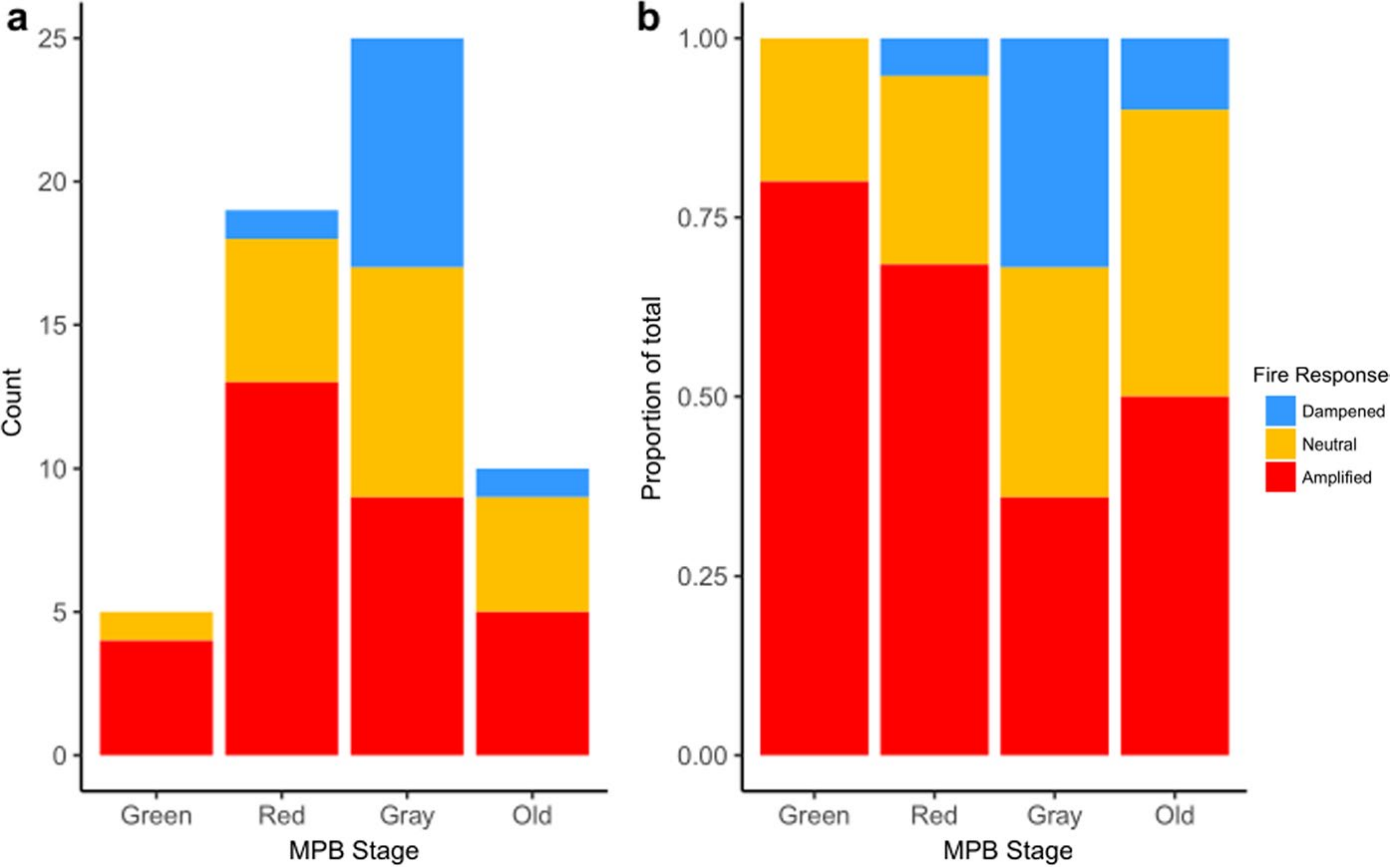
**a** Counts of publication fire responses (n = 35) within each time-since MPB stage across all fire metrics (see text for details). Stages include green (0–1; unattacked to year of attack), red (1–2 years post-outbreak), gray (3–10 years post-outbreak), and old (10 + years post-outbreak). *See text for details*. **b** Proportional counts of publication fire response within each time-since MPB stage across all fire metrics

Of the 27 publications, seven contained multiple fire responses for a total of 35 fire responses. The majority of studies included gray-stage stands or individuals (*n* = 25/35), most included red-stage stands or individuals (*n* = 19/35), whereas only some included green (*n* = 5/35) or old (*n* = 10/35). Proportionately, 80% of studies that measured green-stage MPB outbreak concluded an amplified fire response (i.e., *n* = 4/5 responses), similarly ~ 68% of studies including red-stage outbreak concluded an amplified fire response with the majority of remaining responses attributed to a neutral fire response (Fig. 3a, b). Conversely, gray-stage outbreak was split almost equally between fire responses (dampened 32%, neutral 32%, and amplified 36%). Further, old-stage outbreak was divided almost equally between amplified (50%) and neutral (40%) fire responses, totaling 90% **(**Fig. 3b**)**.

To ensure a good sample size per category and enable comparisons among studies we created a secondary database including only those studies assessing fire intensity and/or fire severity (*n* = 19), noting that some studies use intensity and severity interchangeably. Hence, studies which examined fuel flammability (e.g., time to ignition; *n* = 5), or fire characteristics (area burned; *n* = 2, presence/absence of fire; *n* = 4) were excluded from this dataset.

**Fig. 4:**
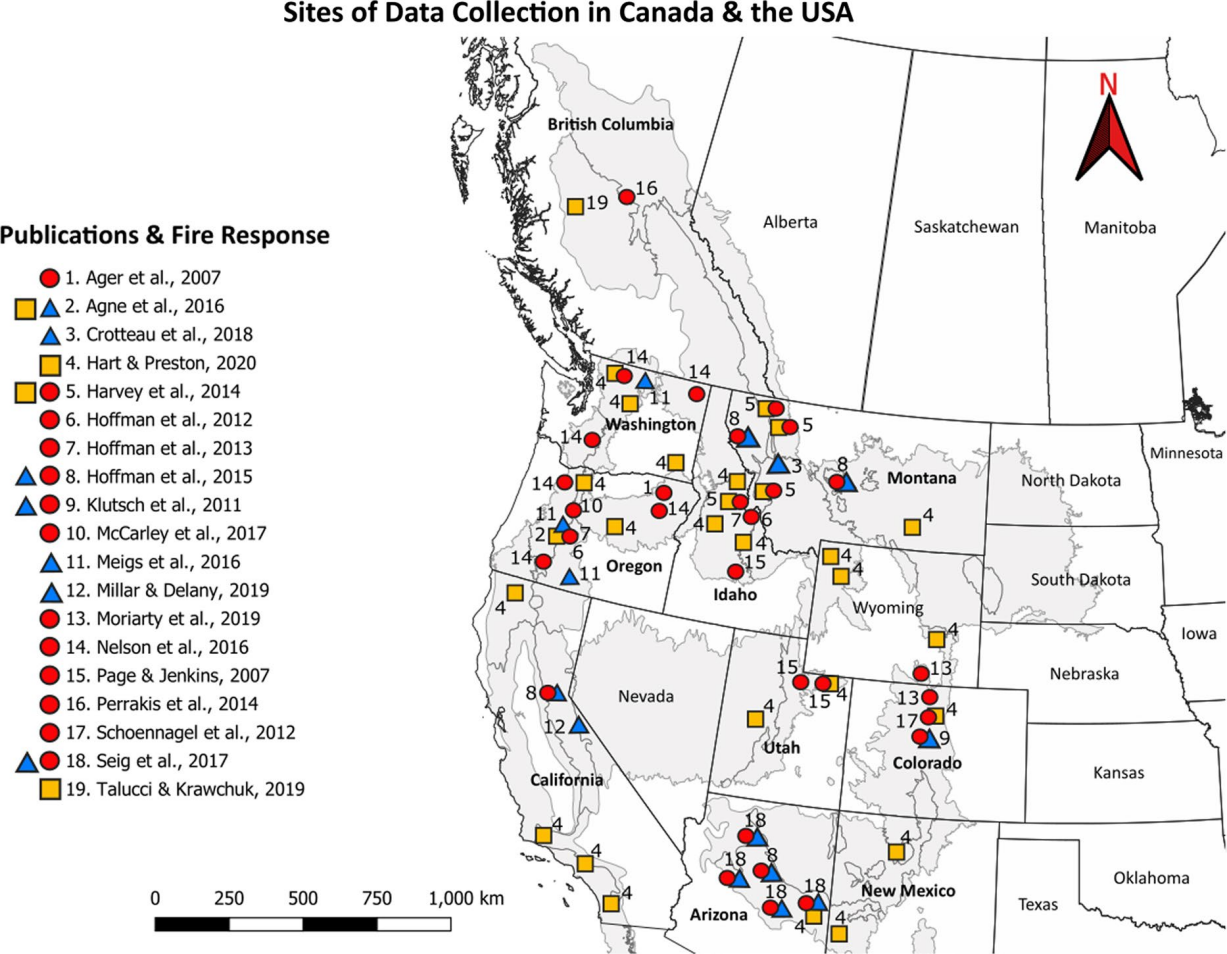
Publication sites of data collection across western Canada, and the USA. Red (circle), yellow (square), and blue (triangle) points represent amplified, neutral, and dampened fire responses. Point locations correspond to the coordinates of publication data collection and are labeled according to publication number. Gray areas indicate pertinent ecoregions containing MPB host trees

This secondary database included a total of 19 publications and 24 fire severity and intensity responses (given that five publications have multiple responses; Fig. S1). Of the 24 responses, seven concluded that MPB outbreak had a dampening effect on fire severity or intensity, four found neutral effects, and 13 reported amplifying effects (Table S2). These studies were conducted across the full latitudinal range of the study area (Fig. 4). We further categorized papers based on the dominant form of data collection, either through “measurement” (e.g., empirical data collection), or “modeling” (e.g., use of numerical/simulation models) (Fig. S1). Studies measuring or modeling fire *severity* were split equally amongst the three response categories. However, studies measuring or modeling fire *intensity* were heavily skewed towards the amplified response category (Fig. S1). This finding was mainly driven by the disproportionate number of studies modeling fire intensity (ten) compared to measuring fire intensity (three).

### Unsupervised classification of fire responses

Publications in the secondary database were charted according to 14 categories of indicators for a total of 48 indicators (Table 1). We then conducted a global principal component analysis (PCA) to analyse the association between each publication’s conclusion(s) of wildfire severity or intensity and the various methodological and respective environmental variables examined.

Using the Broken-Stick criterion (Peres-Neto et al. 2003), we identified the first four principal component (PC) axes as significant, which accounted for 56.06% of the total variation (Fig. S2). The first and second PC axes explained 34.33% of the total variation in our data; thus, to better understand the drivers of these first two significant axes we also used the Broken-Stick criterion to identify significant associations between these axes and the indicator loadings (Peres-Neto et al. 2003). We identified 20 significant indicators (see *PCA statistical analysis* within *Methods*) associated with PC1, and 23 significant indicators associated with PC2 (Table 2) with the top 10 significant indicators given in Table S3.

**Table 2:** Significant indicators associated with PC1 and PC2

Category	Significant indicators: PC1	Significant indicators: PC2
Fire metric	Intensity modeled, severity modeled	Intensity modeled
Wildfire fuels	Ladder fuels, canopy fuels, connectivity, drying rate/fuel size, fuel moisture	Surface fuels, canopy fuels, connectivity, drying rate/fuel size, fuel moisture
Spatial scale	Stand, landscape	Plot/individual
Fire weather	Fire weather considered, and: wind speed	Fire weather considered, and: Temperature, relative humidity, precipitation
Fire weather category	Very high/extreme	Moderate
Time-since-beetle stage	Gray	Red, old
Outbreak intensity	N/A	Outbreak low, moderate
MPB range	MPB range included	MPB range included
Tree species	Multiple species, and: Western white pine (*Pinus monticola)*	Multiple species, and: Western white pine (*Pinus monticola);* White bark pine (Pinus albicaulis)
Decade of dataset	< 2000s, 2010s	< 2000s
Geographic division	Western USA multiple, east SW	Western Canada, west NW

**Fig. 5:**
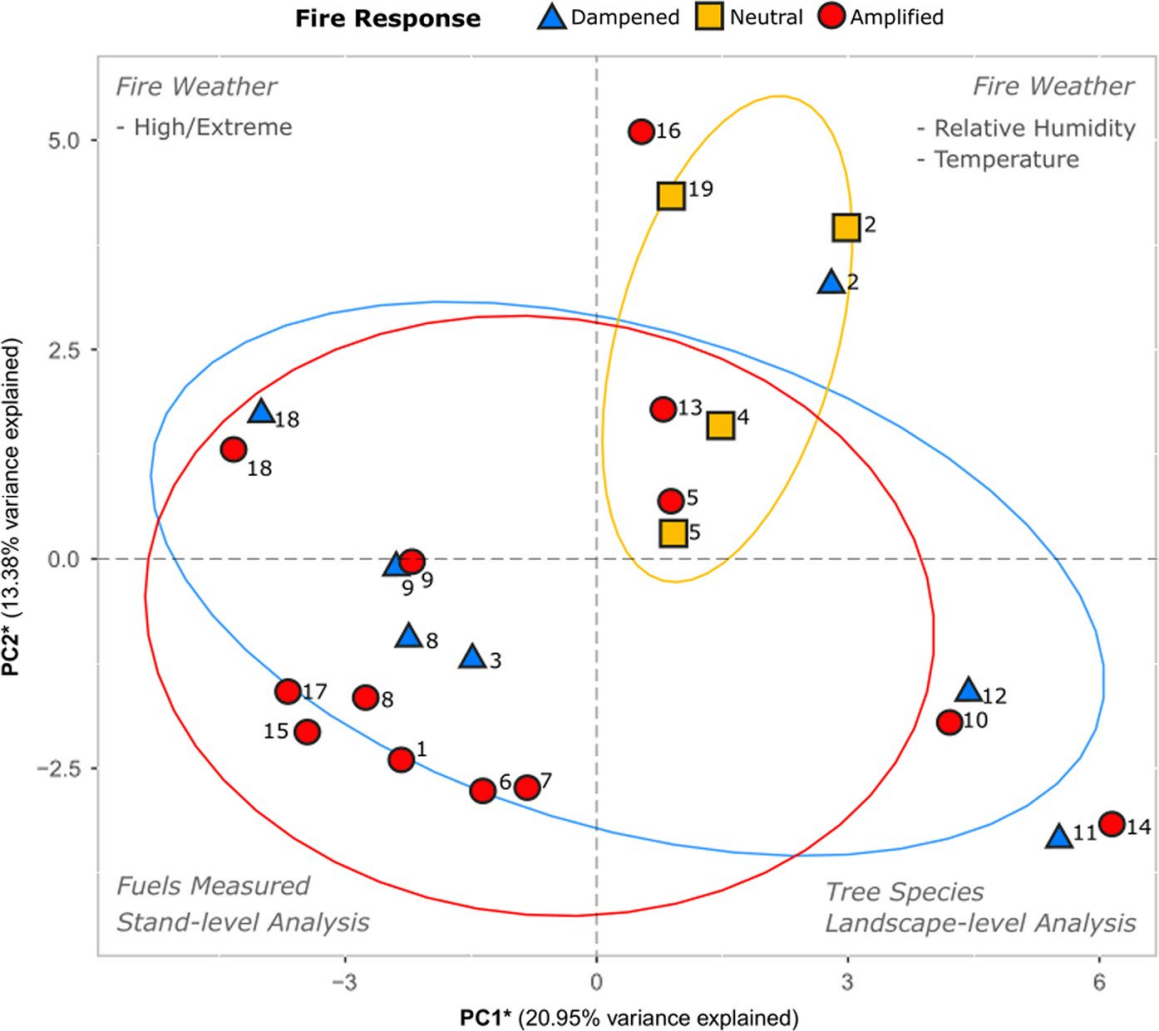
Biplot summarizing the principal component analysis (PCA) of global indicators used to analyse each publication classified into three fire response categories: red—amplified, yellow—neutral, and blue—dampened. *Points* in this biplot represent publications plotted in ordination space and are coordinated to the associated studies’ conclusion(s) regarding fire in response to the indicator variables analysed/measured. Labels in each quadrant represent the most significant indicators driving associated fire responses in the corresponding direction. Ellipses corresponding to each fire response represent the default confidence interval of 68%, and were added to visually separate groups of response observations

PC1 captured a gradient in weather, fuels, species, and scale of analysis (Fig. 5.) The left side of the biplot is associated with fuel attributes, analysis at the stand-scale, fire weather considered, and modeled fire intensity. The right side of the biplot is associated with study timing (i.e., relatively recent—that is, papers published with data collected between 2010 and 2020), consideration of MPB range in analysis, and analysis at the landscape-scale. Most studies that identified an amplified response of wildfire to MPB history were found in the lower left quadrant of the PCA biplot, whereas studies that found a dampened response to MPB activity were more evenly spread across PC1. PC2 captured a gradient in fuels and forest structure and weather, with studies that emphasized fire weather found in the upper half of the ordination biplot and those with greater emphasis on fuels in the lower half. Studies that identified a neutral fire response align strongly with PC2 (Fig. 5). Studies with amplified and dampened responses did not exhibit much variation along PC2, with a few exceptions (e.g., studies 16 and 2). Overall, we were not able to strongly distinguish dampened from amplified wildfire responses to MPB outbreaks in this ordination space except for the cluster of amplified responses in the lower-left quadrant. Neutral responses exhibited a pattern that was distinct from the amplified and dampened responses and appear associated with the explicit inclusion of weather.

### Supervised classification of fire responses

**Fig. 6:**
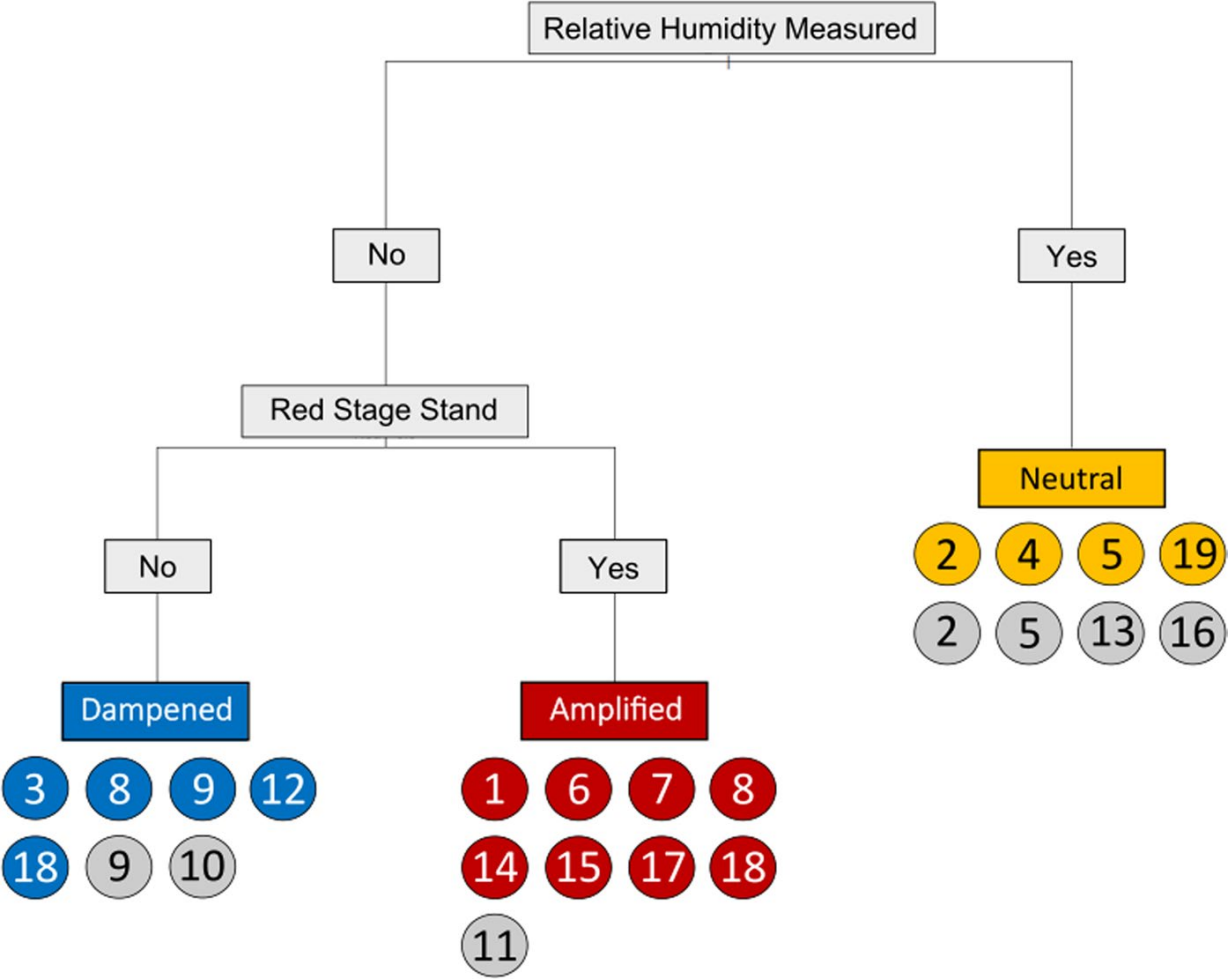
Classification tree model using filtered indicators with Pearson correlation coefficient threshold of 0.8. Publications are grouped below each fire response label and are labeled according to the list of papers outlined in Fig. 4. Blue, red, and yellow coloured circles represent publications that are correctly classified per group, whereas gray coloured circles represent misclassified publications. Consideration of Relative Humidity was identified as the most important indicator in predicting fire response. For studies that include relative humidity in their analyses, the predicted fire response outcome is neutral whereas studies that do not consider relative humidity tended to be associated with an amplified or dampened response. Studies that did not consider relative humidity, but did examine red-stage MPB stand tended to exhibit amplified fire severity responses. Studies that considered neither relative humidity nor red-stage stands tended to exhibit dampened fire severity responses

The multi-class classification tree developed using the Classification and Regression Trees (CART) method (Ripley 2022) had a total of three terminal nodes with an overall accuracy of 0.71 and a misclassification rate of 0.29 (Fig. 6). Overall precision and recall were both 0.933. The first split was conducted on the indicator *relative humidity* which refers to the presence/absence of a measure of relative humidity in the study. The second split followed from relative humidity < 0.5 (i.e., relative humidity = 0 since this was a binary predictor). The split was conducted using the indicator *red stage* where studies of red-stage stands were classified as amplified fire response and those not of red-stage stands were classified as dampened fire response. Note that none of the remaining indicators were significant in the model. At the class (response) level precision was lowest for neutral at 0.5 (i.e., there were equal numbers of true and false positives), while recall was highest at 1.00 (i.e., there were no false negatives). Precision was highest for amplified fire responses at 0.889, but recall was lowest at 0.667. For dampened responses precision was 0.714 and recall was 0.882 (Tables S5-S6).

## Discussion

We found little consensus throughout the literature regarding how MPB outbreaks affect subsequent fires (Figs. 3a & b, 4, 5), despite these interactive forest disturbances being one of the most well-studied in North America (Canelles et al. 2021; Fettig et al. 2021). We hypothesized that studies assessing red-stage stands, and studies conducted under low-moderate fire weather, would be strongly associated with an amplified fire response, whereas studies under extreme fire weather were expected to conclude a neutral fire response. We also expected studies assessing gray-stage stands, and those at the landscape-scale would be associated with a dampened fire response. While we found that MPB outbreak stage (time-since-beetle) was an important driver of fire response (Figs. 5, 6), as were measures of wildfire fuels (Fig. 5), we also found unexpected associations with weather (Figs. 5, 6), and a non-significant influence of research method on fire response. In general, studies did not examine the possible role of within-fire spatial variation in MPB outbreak intensity on burn severity. Given the large spatial scales over which MPB outbreaks occur, most fires examined and reported upon in the literature we reviewed occurred within a relatively homogenous MPB-affected landscape or stand. However, opportunity remains to further refine our understanding of how finer-scale heterogeneity in outbreak attributes can influence wildfire behaviour and outcomes.

The lack of consensus in the literature may, in part, be explained by variations in experimental and methodological decisions made among studies. Specifically, no standardized protocol exists to measure environmental variables within MPB-affected stands for observational/empirical studies (e.g., forest inventory and fire weather metrics—see indicators in Table 1). Therefore, we suggest the implementation of predefined metrics for use in future analyses similar to the system used by the CBI (Key and Benson 2006). Such a protocol would lead to consistent methods of data collection among future studies and facilitate the easier comparison of results across studies. Further, we found that the spatial scale used for analysis is highly variable as studies range from individual plot (Agne et al. 2016; Sieg et al. 2017; Talucci and Krawchuk 2019) to stand (Page and Jenkins 2007; Schoennagel et al. 2012; Perrakis et al. 2014; Nelson et al. 2016) to landscape-scale (Meigs et al. 2016; McCarley et al. 2017; Hart and Preston 2020), and that this may influence the reported fire response. While these issues are important to resolve for the accurate inclusion of MPB-fire interactions in forest and fire management strategies, the limited consensus in fire response to MPB outbreaks may also be driven by the variation in climate and weather conditions, topography, and historical forest management methods across sites (Stephens et al. 2022; Talucci et al. 2022). Thus, this context- and possible scale-dependency has the potential to overshadow the direct impacts of MPB-outbreaks on subsequent wildfire.

The MPB has recently expanded its range into historically unaffected conifer forests (Safranyik and Carroll 2006; Bentz et al. 2011; Sambaraju et al. 2012). Coupled with predicted climate-driven doubling in area burned in western North America, interactions between these two disturbances are expected to increase. Improved understanding and appropriate management of MPB-attacked forests is critical to mitigate the potential negative effects of this disturbance interaction such as alterations to forest biodiversity and associated ecosystem functions and services via spatial legacies (i.e., physical changes in forest stand, and fuel structures) left behind post-outbreak (Flannigan et al. 2009; Turner 2010). We found that fire intensity and severity can be both increased or decreased by the spatial legacies of MPB outbreaks, and that this effect depends on the time since outbreak and fire weather. We suggest that further resolution of this important interaction can be achieved using a combination of standardized protocols and complementary remote sensing and LiDAR approaches. Together, such approaches hold potential to further improve the reliability and comparability of fire-insect interaction studies and contribute to overcoming challenges related to spatial scale (Key and Benson 2006). Knowledge of how key drivers of fire responses to MPB outbreaks and associated spatial legacies vary through space and time is essential to guide forest and fire management strategies (Liebhold and Bentz 2011; Turner 2005, 2010) in western Canada and the USA, especially in forests that are newly exposed to this disturbance interaction due to climate change.

### Methodology

The majority of papers we examined focused on fire intensity and severity through either direct measurements or modeling methods (*n* = 24/35 unfiltered fire responses). Fire intensity papers mostly used a modeling approach to draw conclusions about MPB-wildfire interactions (Fig. S1) likely due to the difficult to predict and stochastic nature of wildfire ignitions and associated challenges with collecting *in situ* data for fire intensity (Filkov et al. 2018). The potential to measure fire severity both remotely and on the ground after the fire likely accounts for the dominance of direct, empirical measurements in this category (*n* = 9) and resulted in a similar number of studies that modeled fire intensity (*n* = 10). Challenges with *in-situ* data collection and ecologically relevant temporal scales may limit both the amount and breadth of data collected on fire intensity following outbreaks, although high resolution remote sensing data and LiDAR are likely to advance fire severity data availability across all spatial scales (French et al. 2008; Wulder et al. 2009; Alonzo et al. 2017).

### Spatial scale of analysis

The spatial scale of analyses (individual tree/plot, stand, or landscape) used in studies separates fire responses on the PCA but is not the strongest control on the fire response of studies. The three spatial scales assessed were associated with different axes of the PCA (Fig. 5). Stand-scale studies were associated with PC1 and amplified wildfire responses. The use of stand-scale forest fuels data in fire behavior models (Hoffman et al. 2012) may have contributed to this outcome since the majority of modeled fire intensity studies concluded an amplified response (7/10). Contrary to our hypotheses, landscape-scale analyses were associated with both dampened and amplified fire responses (Fig. 5). Landscape-scale analyses typically employed remotely sensed metrics to assess fire severity such as the normalized burn ratio (NBR), delta NBR (dNBR) and relative dNBR (RdNBR) that assess changes in live vegetation cover and associated spectral reflectance (Meigs et al. 2016; McCarley et al. 2017). The resolution of these products (up to 1 km pixels, but typically 30 m when derived from Landsat) may affect the ability to assess fire severity in specific MPB-attacked areas as MPB outbreaks are known to be heterogeneous at a finer spatial scale throughout a stand (Safranyik and Carroll 2006) and often results in spatial clustering (Hoffman et al. 2015). Plot- and stand-scale analyses of fire severity typically employed the composite burn index (CBI; Agne et al. 2016; Millar and Delany 2019; Talucci and Krawchuk 2019) which provides information on specific aspects of fire severity such as char height, bole scorch, surface and canopy fuel consumed, and post-fire litter consumed. The plot-scale indicator was significant on PC2 (associated with a neutral response), while stand-scale was significant on PC1 in the negative loading direction. The divergence of the impacts of experimental design on wildfire response to MPB outbreaks makes it challenging to accurately compare study results to one another. Such challenges may be addressed by integrating across multiple spatial scales, linking field assessments or stand-scale modeling with landscape-scale remote sensing data.

### Forest fuels and MPB stages

Studies that include forest fuel metrics in their analysis were found to be associated with amplified fire severity and intensity responses following an outbreak. Forest fuel metrics such as surface fuel and canopy fuel load, as well as fuel size, are closely linked with the modeled fire intensity and, as mentioned, stand-scale analyses, because estimates of forest fuel loads and moisture content are required by fire behavior models (Wotton 2009). As such, most fire intensity studies found an amplifying effect of MPB on wildfire. In contrast, studies that directly measured fire severity found dampened, neutral, and amplified responses in nearly equal proportions (Fig. S1). While variability in fire response may be due to the context-dependent nature of fire impacts on forests, the varied forest structures following MPB outbreak (Schoennagel et al. 2012) are likely to play a significant role (Jenkins et al. 2014).

The development of MPB outbreaks and subsequent tree mortality affects forest fuels. Here we show that different stages of MPB are predominantly associated with different fire responses. In support of our hypothesis, early-stage outbreaks (e.g., green- to red-stage), where needles are still present in the canopy but moisture levels are reduced (Gray et al. 2021; Jolly et al. 2012; Klutsch et al. 2011; Page et al. 2012), were associated with amplified fire response (Figs. 3, 5). Further, the presence of red-stage outbreak is significant in the classification model (Fig. 6) and associated with amplified fire response (Fig. 5, 6). Conversely, gray-stage outbreak is the only stage to have approximately equal representation of dampened, neutral, and amplified fire responses and is more closely associated with a neutral response on the PCA (Fig. 3, 5). The limitation of fire intensity or severity in gray-stage stands is often attributed to the reduction in canopy fuel load (Klutsch et al. 2009; Meigs et al. 2016; Simard et al. 2011). However, the effect of wind channeling in gray-stage stands may have important implications for fire behavior and fire suppression (Hoffman et al. 2015).

This finding is also likely attributed to the ways in which studies assess fire severity. Despite, analysing factors such as deep char, char height, charred surface, and bole scorch (etc.) methodological practices, and the location/geography of a study, play key roles in the quantification of fire severity post-MPB outbreaks. For instance, Talucci and Krawchuk (2019) found that majority of the fire effects analysed in their study—that is, scorch and deep charring—were not influenced by MPB outbreak severity; rather, fire effects were more strongly influenced by fuel structure, weather, and topography. Similarly, Talucci et al. (2022) also found that outbreak severity alone did not influence fire severity such that peak high-severity fires were associated with the spatial legacies left by previous MPB outbreaks (i.e., dead trees and biomass) combined with live forest vegetation. Further, Harvey et al. (2014a) note a decrease in canopy fire severity in gray-stage stands under moderate burning conditions, and no effect under extreme burning conditions, whereas surface fire severity is unrelated to MPB outbreak severity in the gray-stage.

Old-stage outbreak was significantly associated with the second PC axis (Fig. 5) and is associated with mixed fire responses; half of the recorded responses were amplified while the remainder were split between dampened and neutral (Fig. 3). Due to the lack of an upper limit of time-since-beetle included in this category, there is potential for the influence of regenerating vegetation on wildfire to be measured in some studies and not others.

Despite the variation within categories, the conceptualization of changing fire outcomes from different time-since MPB outbreak stages could help develop post-fire management plans for fires of different severities, based on their time-since outbreak at time of ignition. While there is also potential to help firefighters avoid “surprising” wildfire behavior in different time-since outbreak stands, responders interviewed in Moriarty et al. (2019) suggested there was less difference between fire behavior in red- and gray-stage stands than they had expected, with both showing relatively high fire intensity.

Differences in definitions used across studies may, in part, further explain contradictory wildfire responses. Two studies that conclude amplified fire responses to MPB in later-stage stands (Harvey et al. 2014a, b; Page and Jenkins 2007) assess a range of fire severity and intensity metrics and their conclusions of amplified fire are mostly attributed to surface fire behavior. This is in contrast to crown fire behavior, which is assessed in other studies (Hoffman et al. 2012). The seemingly subtle difference in fire metric analysed may change the reported “direction” of the disturbance interaction and highlights the need for specificity when reporting changes to wildfire. Further, the interchangeable use of fire severity and fire intensity, which has been troubling fire science for decades (see Keeley 2009; Key and Benson 2006), may further distort the true direction or magnitude of disturbance interactions.

### Fire weather

Fire severity and intensity are inextricably linked to fire weather, characterized typically by measures of temperature, precipitation, relative humidity and wind speed (Stocks et al. 1989). The importance of fire weather for fire intensity and severity is supported by our PCA where wind speed had the highest loading on PC1 and relative humidity and temperature had the two highest loadings on PC2 (Table S3). Further, we identified relative humidity as a significant predictor of fire response (Fig. 6), indicating that studies which explicitly considered weather variables in their analyses were more likely to find a neutral or non-significant effect of MPB on subsequent wildfire activity. Contrary to our hypothesis, studies that specifically documented high or extreme fire weather were not more associated with neutral (non-significant) fire responses following MPB outbreak. While we hypothesized that extreme fire weather would override the impact of MPB spatial legacies on fire, there may be an interaction between aspects of extreme fire weather that amplify changes in wildfire fuels caused by MPB outbreak such as flammability (Jolly et al. 2012; Page et al. 2012), reduced fuel moisture (Jolly et al. 2012; Page et al. 2012; Gray et al. 2021) and/or altered in-stand micrometeorological conditions (Hoffman et al. 2012), leading to higher intensity fires across a range of outbreak stages (Moriarty et al. 2019). More work is needed to disentangle these complex cross-scale interactions (Peters et al. 2004). For instance, wildfire activity within a MPB affected stand can be influenced (i.e., amplified or dampened) as a result of positive or negative feedbacks between fire weather and the spatial legacies left behind by MPBs in forest stand and fuel structure. Harvey et al. (2014a) is one of the few studies which examined the interacting effects of different MPB outbreak stages under different weather conditions. Their study identified six unique fire severity responses wherein moderate burning conditions led to amplified fire severity in green- and red-stage stands, but dampened severity in gray-stage stands. Furthermore, there was no effect on fire severity across green-, red-, and gray-stage stands under extreme burning conditions. Such mixed results reported in Harvey et al. (2014a) supports our conclusion that fire response post-MPB outbreak largely depends on environmental conditions such as the number of years since an outbreak (i.e., TSB stage) and fire weather conditions.

The occurrence of a positive interactions between high-extreme fire weather and MPB outbreak stages will need to be tested by future research to best inform fire management agencies that will be managing fires under more extreme fire weather conditions due to climate change (Bentz and Klepzig 2014; Flannigan et al. 2009). Further, research is required to better understand the drivers of this interaction outcome which may range from increased fuel availability due to lower fuel moisture coupled with high ignition potential in MPB-attacked stands (Gray et al. 2021; Jolly et al. 2012; Page et al. 2012), to increased wind speeds promoting higher rates of spread, compounded with increased wind channeling in gray-stage, more open, stands (Hoffman et al. 2015; Page and Jenkins 2007). Moreover, the potential for more intense droughts or windstorms driven by climate change should be considered through their impact on the spatial legacies of MPB outbreak and subsequent impacts on wildfire (Liebhold and Bentz 2011).

### Geographic division

Despite the vast geographic extent of the studies we examined, neither ecoregion nor geographic division strongly influenced fire response, although replicates per ecoregion category were typically low. This finding suggests that local climate, topography, elevation, prior forest management, and post-fire stand regeneration will need to be considered when assessing MPB-wildfire interactions in existing and newly attacked areas; general conclusions and uniform rules governing these interactions seem unlikely. These factors influence forest structure and fuel loadings, and impact susceptibility to other disturbances such as drought, which can increase the vulnerability to both MPB-attack and wildfire (Liebhold and Bentz 2011).

Forested landscapes in BC, Canada, and western USA states have distinct characteristics that influence interactions between MPB outbreaks and wildfire (Raffa et al. 2008). Specifically, these regions vary in terms of forest composition, outbreak extent, MPB host tree availability, tree defensive ability, and nutritional quality necessary to support outbreaks (Meddens et al. 2012; Wulder et al. 2010; Talucci et al. 2022). Variation in these attributes is shaped by previous disturbances, weather and climate, topography, and soil characteristics (Raffa et al. 2008).

Differences in weather and climatic conditions—specifically, temperature and precipitation—between BC and western USA states also influence the extent of MPB outbreaks through the relationship between beetles and their host trees (Raffa et al. 2008). MPB life history traits (i.e., reproduction, development and survival of larvae, and voltinism) and outbreak dynamics (i.e., flight time and duration) are strongly governed by temperature. Additionally, host trees, such as lodgepole pine, are vulnerable to changes in precipitation wherein severe drought can reduce tree defenses against MPB mass attack (Raffa et al. 2008).

We did identify some support of our geographic hypothesis in that studies from the western USA were more often associated with an amplified fire response and those in Canada were more often associated with a neutral fire response. However, conclusions in this regard are hampered by a small sample size for Canadian studies (*n* = 2/19 secondary database). This highlights a critical need for more focused MPB—wildfire research in Canadian forests, especially given record-breaking MPB outbreaks in British Columbia (Natural Resources Canada 2021) and its spread to neighboring Alberta and the Yukon (Cooke and Carroll 2017), as well as with record-breaking fire seasons in recent years (Natural Resources Canada 2021). 

## Conclusions

This meta-analysis aimed to enhance the state of understanding of the interaction between MPB outbreaks and wildfire in Canada and the USA through a scoping review. Many of the studies we examined suggest that MPB outbreaks influence wildfires through the legacies they leave behind in the form of forest stand and fuel structures. Through our review of the literature, we found substantial evidence that time-since MPB stage is indeed associated with different fire responses. Red-stage stands were were associated with an amplified fire response, while green-, gray- and old-stage stands were associated with a neutral or dampened fire response. However, MPB-stage does not explain all the variability in fire responses. Spatial scale, fuels measurement, and weather conditions also seem to affect our ability to identify meaningful effects of MPB outbreaks on subsequent wildfire severity and intensity.

Interactions among disturbances, including those between wildfire and insect outbreaks, can lead to landscape scale changes in forest ecosystems structure and function. Inferences regarding the character of these interactions in one forest ecosystem are not necessarily applicable to forests elsewhere. Therefore, environmental conditions unique to forest ecosystems (i.e., weather conditions, species composition, stand and fuel structure, topography, etc.), should be considered when investigating similar disturbance interactions in different regions.

## Supplementary Information

Below is the link to the electronic supplementary material.

Supplementary file1 (PDF 615 KB)

## Data Availability

The datasets and code analysed during and/or generated during the current study are available from the corresponding author on reasonable request.
