# Supplementary material for: On the limited consensus of mountain pine beetle impacts on wildfire
Source: Landsc Ecol. 2023 Jul 13;38(9):2159–78. doi: 10.1007/s10980-023-01720-z (PMC10372117; doi:10.1007/s10980-023-01720-z)
Supplement: Supplementary file 1 — Supplementary file1 (PDF 615 KB) [file 10980_2023_1720_MOESM1_ESM.pdf]

Landscape Ecology

On the Limited Consensus of Mountain Pine Beetle Impacts on Wildfire

D. C. Romualdi*[1], S. L. Wilkinson[1], and P. M. A. James[1]

[1]Institute of Forestry and Conservation, Daniels Faculty of Landscape, Architecture and Design, University of Toronto, Canada.

*Corresponding author – D. C. Romualdi

Address: 33 Willcocks St, Toronto, Ontario, M5S 3B3

Email: doriana.romualdi@mail.utoronto.ca

Telephone: (647)-889-4501

ORCID:

DCR 0000-0001-9042-0882

SLW 0000-0002-4043-6277

PMAJ 0000-0001-7639-7217

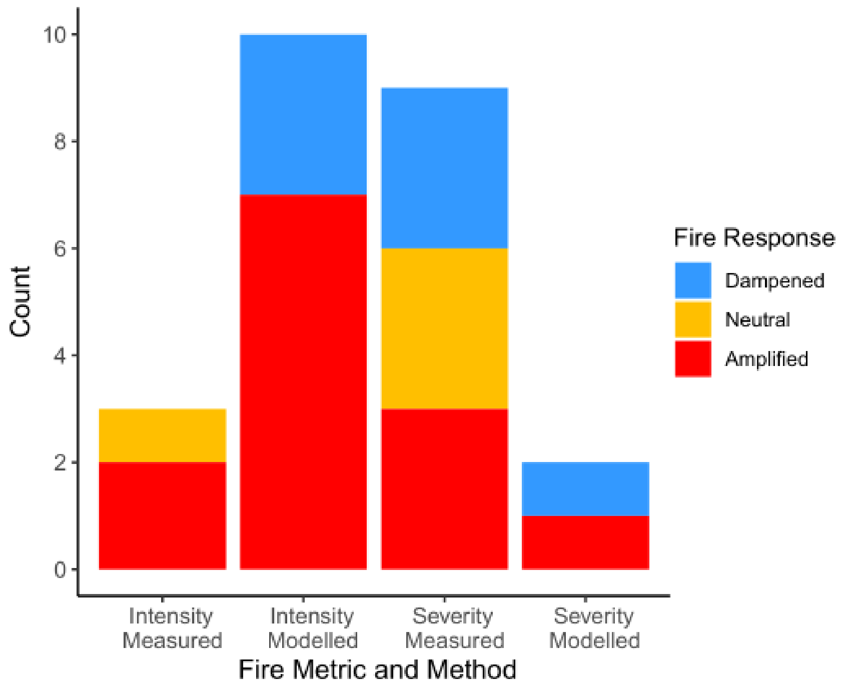

Figure S1 Counts of papers per fire metric and method in the filtered (secondary) database focusing only on severity and intensity. Colors represent the fire response wherein blue, yellow, and red correspond to dampened, neutral, and amplified responses, respectively

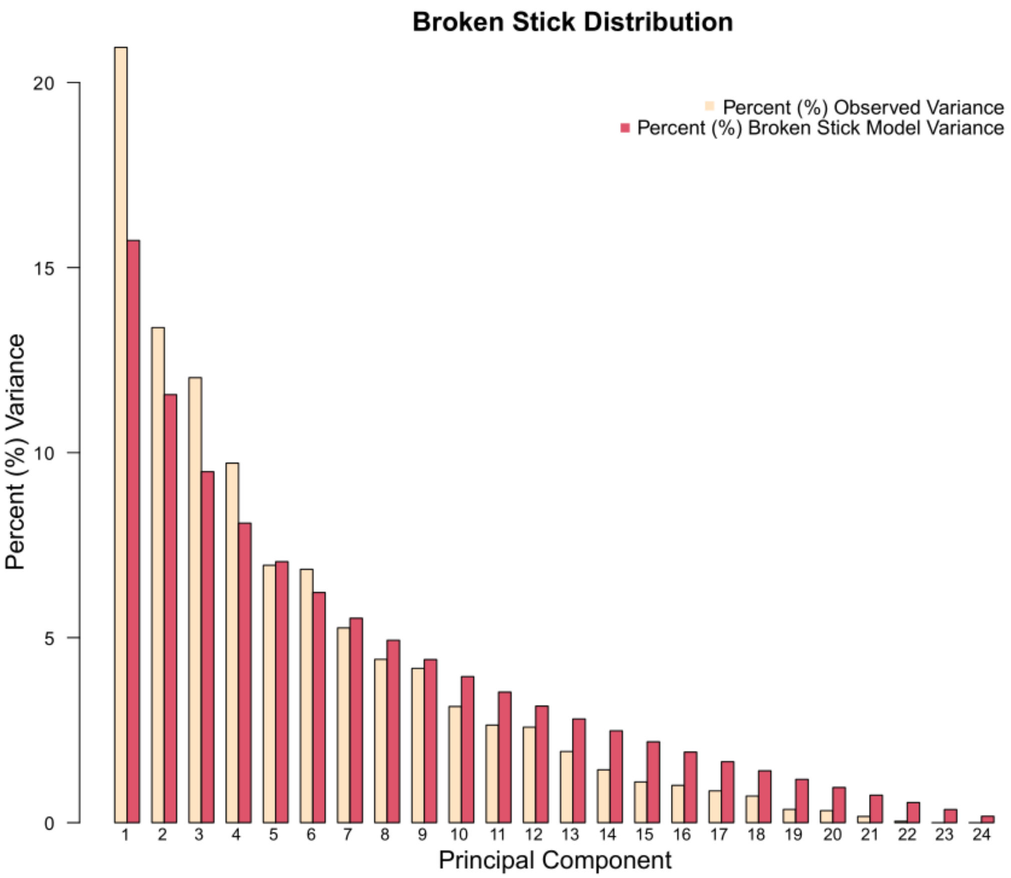

Figure S2 Broken stick criterion showing significant PCA axes where the proportion of observed variance explained by the data is greater than the expected proportion of variance under the Broken-Stick model

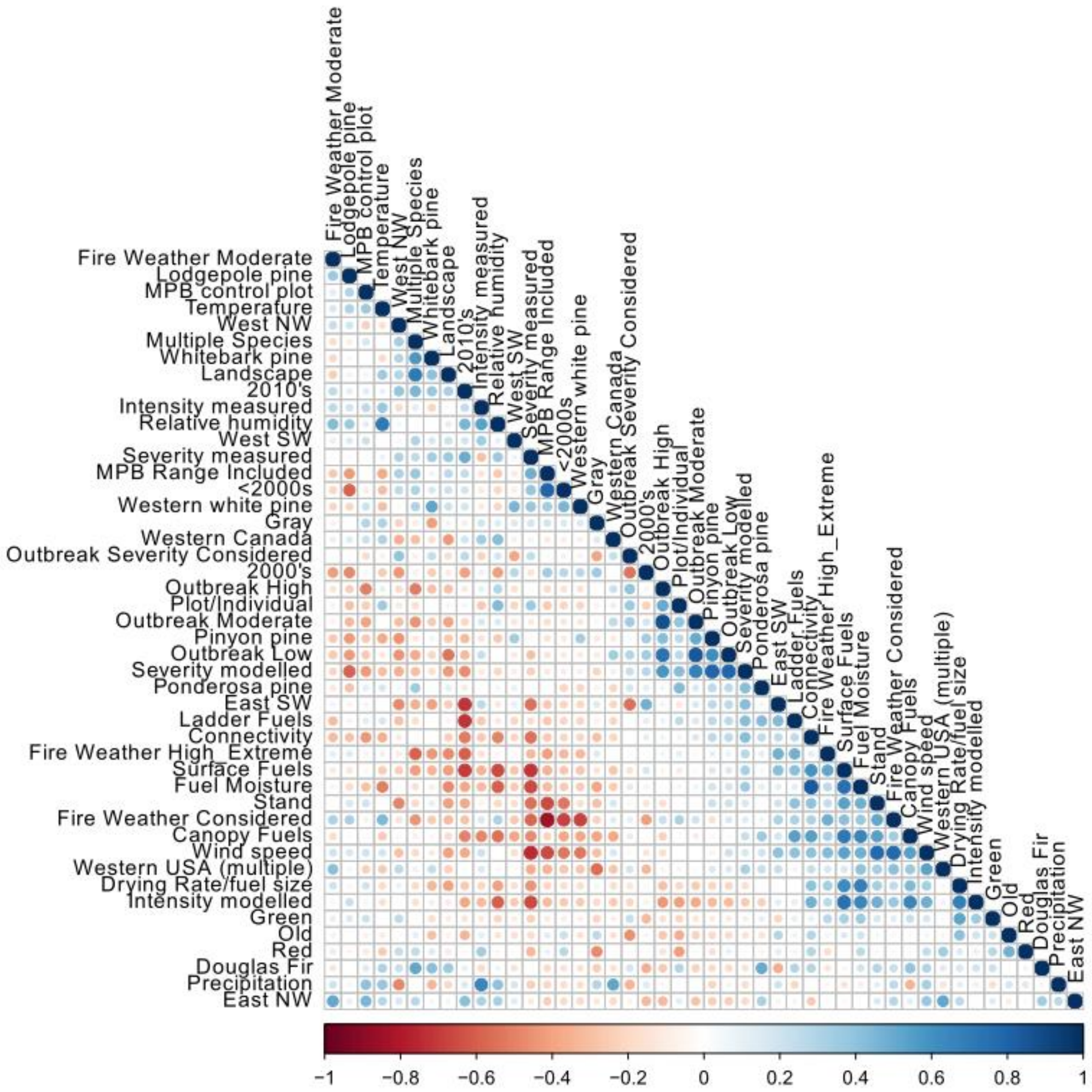

Figure S3 Correlation plot of all indicators generated using the *corrplot* function in the corrplot package on a correlation matrix created using the *cor* function in the **corrgram** package. Pearson correlation coefficients greater than zero are blue in color and represent a positive correlation, whereas coefficients less than zero are red in color and represent a negative correlation - both becoming darker in their respective shade closer to each extreme end (i.e., -1 and 1). Correlation coefficients of zero are coloured white

Table S1. List of publications excluded from the secondary database used in statistical analyses.

**Publications Excluded from Secondary Database**	**Justification**
Bourbonnais et al. (2014)	Analysed fire ignition density in response to MPB severity and TSB stage
Hart et al. (2015)	Analysed total annual area burned in response to cumulative area of MPB outbreak
Hart and Preston (2020)	Analysed daily area burned in response to MPB outbreaks
Jolly et al. (2012a)	Analysed the relationship between foliar moisture and chemistry, and fire ignition in early stages of MPB attack
Jolly et al. (2012b)	This publication is a comment on Simard et al. (2011)
Kulakowski and Jarvis (2011)	Analysed the influence of historical MPB outbreaks on more general fire regime traits (i.e., fire occurrence, area burned, and risk)
Lynch et al. (2006)	Analysed fire risk in response to MPB activity
Meigs et al. (2015)	Analysed fire likelihood following insect outbreaks
Mietkiewicz and Kulakowski (2016)	Analysed annual area burned and fire perimeter in response to MPB outbreaks
Page et al. (2012)	Analysed fuel chemistry and flammability in MPB attacked lodgepole pine forests
Simard et al. (2011)	Although this publication examined the effects of MPB outbreak severity on fire behaviour characteristics including fireline intensity, the comment by Jolly et al. (2012b) highlights multiple reasons as to why the conclusions outlined in this publication are compromised.<br><br>Jolly et al. (2012b) suggest that the fire behaviour modeling frameworks used by Simard et al. (2011), the NEXUS crown fire behaviour modeling system, is unsuitable to properly assess MPB-fire interactions. This is mainly due to the assumptions of this modeling system being homogenous, continuous forest fuels, and steady-state flame spread. Such fuel and flame spread assumptions are inapplicable to stands attacked by MPB wherein fuels are highly heterogeneous in their continuity, and moisture content.

	Further, Jolly et al. (2012b) explain that Simard et al.'s (2011) use of this crown fire modeling framework omits important details relating to alterations in canopy and surface fuels in stands recently (i.e., red-stage) attacked by MPB. Specifically, they outline how Simard et al. (2011) do not properly characterize red-stage stand canopy bulk density, and foliar moisture content – both of which have the potential to influence crown fire activity. Further, Simard et al. (2011) also omit changes in litter depth in early attack stage stands to predict surface fire intensity. Lastly, Jolly et al. (2012b) emphasize how Simard et al.'s (2011) use of a space-for-time chronosequence sampling method resulted in high fuel variability among time-since-beetle stages which led to an inaccurate effect of MPB outbreaks over time.<br><br>For these reasons, Simard et al. (2011) predicted a decrease in crown fire potential in red-stage stands whereas Jolly et al (2012b) demonstrate how more appropriate estimates of canopy and surface fuels would have led to an increase in crown fire potential within the first three years following an outbreak.
Harvey et al. (2014a)	This publication provides a comprehensive analysis of the effects of MPB on fire severity across green-, red-, and gray-stages under different fire weather conditions. However, it was not included in our meta-analysis primarily because of the range of fires responses that were included. We restricted publications to those with a maximum of two fire severity/intensity responses. Our method of classifying articles does not effectively support the inclusion of studies with more than two fire responses. When multiple (i.e., >2) responses are included, but the rest of the classification information remains the same, many indicators are duplicated and a single study can come to dominate the ordination.<br><br>Harvey et al. (2014a) identified six unique fire responses based on combinations of time-since-beetle (TSB) and fire weather wherein all three fire responses (i.e., dampened, neutral, and amplified) were present. In this case, the only differences in their indicators were TSB (i.e., green, red, and gray) and fire weather (i.e., moderate and extreme). All other indicators were identical. Inclusion of these different responses with identical indicators in our PCA resulted in the ordination being dominated by a cluster of these six observations and reduced our ability to tease apart differences among the other studies included.

	Indeed, these 6 responses would represent 25% (n = 6/24) of our total study responses which explains its ability to overwhelm and obscure other patterns or grouping in our data.<br><br>That said, the mixed results reported in Harvey et al. (2014a) study supports our conclusions from our meta review that fire response post-MPB outbreak depends on environmental conditions such as the number of years since an outbreak (TSB stage) and fire weather conditions.

Table S2. Alphabetically ordered reference papers and associated ID number, and fire response(s).

Paper ID Number	Reference	Concluded Fire Response(s)
1	Ager et al. (2007)	Amplified (+)
2	Agne et al. (2016)	Dampened (-) and neutral (0)
3	Crotteau et al. (2018)	Dampened (-)
4	Hart and Preston (2020)	Neutral (0)
5	Harvey et al. (2014)	Neutral (0), and amplified (+)
6	Hoffman et al. (2012)	Amplified (+)
7	Hoffman et al. (2013)	Amplified (+)
8	Hoffman et al. (2015)	Dampened (-) and amplified (+)
9	Klutsch et al. (2011)	Dampened (-) and amplified (+)
10	McCarley et al. (2017)	Amplified (+)
11	Meigs et al. (2016)	Dampened (-)
12	Millar and Delany (2019)	Dampened (-)
13	Moriarty et al. (2019)	Amplified (+)
14	Nelson et al. (2016)	Amplified (+)
15	Page and Jenkins (2007)	Amplified (+)
16	Perrakis et al. (2014)	Amplified (+)
17	Schoennagel et al. (2012)	Amplified (+)
18	Sieg et al. (2017)	Dampened (-) and amplifies (+)
19	Talucci and Krawchuk (2019)	Neutral (0)

S3. Top 10 indicator loadings for PC1 and PC2.

**Top 10 Significant Indicators: PC1**	PC1 Loadings	PC1 Squared Loadings	**Top 10 Significant Indicators: PC2**	PC2 Loadings	PC2 Squared Loadings
**1. Wind speed**	-0.2643	0.0699	**1. Relative humidity**	0.3262	0.1064
**2. 2010s**	0.2480	0.0615	**2. Temperature**	0.2646	0.0700
**3. Fuel moisture**	-0.2363	0.0558	**3. Western Canada**	0.2379	0.0566
**4. Canopy fuels**	-0.2211	0.0488	**4. Plot individual**	0.2350	0.0552
**5. Stand**	-0.2205	0.0486	**5. Outbreak moderate**	-0.0130	0.0545
**6. Fire weather considered**	-0.2198	0.0483	**6. Multiple species**	-0.2137	0.0456
**7. MPB range included**	0.2106	0.0443	**7. Intensity modeled**	-0.2021	0.0408
**8. Landscape**	0.2058	0.0423	**8. Fire weather considered**	0.2016	0.0406
**9. Intensity modeled**	-0.2049	0.0419	**9. Canopy fuels**	-0.1818	0.0330
**10. Connectivity**	-0.1999	0.0399	**10. Outbreak low**	-0.1377	0.0312

Table S4. Confusion matrix for the results of the multi-class classification tree.

Predicted	Actual		
	**Dampened**	**Neutral**	**Amplified**
**Dampened**	5	0	2
**Neutral**	1	4	3
**Amplified**	1	0	8

Table S5. Performance metrics per response class for the multi-class classification tree.

Performance Metric	Class		
	Dampened	Neutral	Amplified
**Sensitivity**	0.7143	1.0000	0.6154
**Specificity**	0.8824	0.8000	0.9091
**Precision**	0.7143	0.5000	0.8889
**Recall**	0.8824	1.0000	0.6667

SI References

Ager, A. A., McMahan, A., Hayes, J. L., & Smith, E. L. (2007). Modeling the effects of thinning on bark beetle impacts and wildfire potential in the Blue Mountains of eastern Oregon. *Landscape and Urban Planning*, *80*(3), 301-311.

Agne, M. C., Woolley, T., & Fitzgerald, S. (2016). Fire severity and cumulative disturbance effects in the post-mountain pine beetle lodgepole pine forests of the Pole Creek Fire. *Forest Ecology and Management*, *366*, 73-86.

Bourbonnais, M. L., Nelson, T. A., & Wulder, M. A. (2014). Geographic analysis of the impacts of mountain pine beetle infestation on forest fire ignition. *The Canadian Geographer/Le Géographe canadien*, *58*(2), 188-202.

Crotteau, J. S., Keyes, C. R., Hood, S. M., Affleck, D. L., & Sala, A. (2018). Fuel dynamics after a bark beetle outbreak impacts experimental fuel treatments. *Fire Ecology*, *14*(2), 1-17.

Hart, S., & Preston, D. (2020). Fire weather drives daily area burned and observations of fire behavior in mountain pine beetle affected landscapes. *Environmental Research Letters*, *15*(5), 054007.

Hart, S. J., Schoennagel, T., Veblen, T. T., & Chapman, T. B. (2015). Area burned in the western United States is unaffected by recent mountain pine beetle outbreaks. *Proceedings of the National Academy of Sciences*, *112*(14), 4375-4380.

Harvey, B. J., Donato, D. C., Romme, W. H., & Turner, M. G. (2014a). Fire severity and tree regeneration following bark beetle outbreaks: the role of outbreak stage and burning conditions. *Ecological Applications*, *24*(7), 1608-1625.

Harvey, B. J., Donato, D. C., & Turner, M. G. (2014). Recent mountain pine beetle outbreaks, wildfire severity, and postfire tree regeneration in the US Northern Rockies. *Proceedings of the National Academy of Sciences*, *111*(42), 15120-15125.

Hoffman, C., Morgan, P., Mell, W., Parsons, R., Strand, E. K., & Cook, S. (2012). Numerical simulation of crown fire hazard immediately after bark beetle-caused mortality in lodgepole pine forests. *Forest Science*, *58*(2), 178-188.

Hoffman, C. M., Linn, R., Parsons, R., Sieg, C., & Winterkamp, J. (2015). Modeling spatial and temporal dynamics of wind flow and potential fire behavior following a mountain pine beetle outbreak in a lodgepole pine forest. *Agricultural and Forest Meteorology*, *204*, 79-93.

Hoffman, C. M., Morgan, P., Mell, W., Parsons, R., Strand, E., & Cook, S. (2013). Surface fire intensity influences simulated crown fire behavior in lodgepole pine forests with recent mountain pine beetle-caused tree mortality. *Forest Science*, *59*(4), 390-399.

Jolly, W. M., Parsons, R., Varner, J. M., Butler, B. W., Ryan, K. C., & Gucker, C. L. (2012b). Do MPB outbreaks change the probability of active crown fire in lodgepole pine forests? Comment. *Ecology*, *9*(4), 941-946.

Jolly, W. M., Parsons, R. A., Hadlow, A. M., Cohn, G. M., McAllister, S. S., Popp, J. B., Hubbard, R. M., & Negron, J. F. (2012a). Relationships between moisture, chemistry, and ignition of Pinus contorta needles during the early stages of mountain pine beetle attack. *Forest Ecology and Management*, *269*, 52-59. https://doi.org/10.1016/j.foreco.2011.12.022

Klutsch, J. G., Battaglia, M. A., West, D. R., Costello, S. L., & Negrón, J. F. (2011). Evaluating potential fire behavior in lodgepole pine-dominated forests after a mountain pine beetle epidemic in north-central Colorado. *Western Journal of Applied Forestry*, *26*(3), 101-109.

Kulakowski, D., & Jarvis, D. (2011). The influence of mountain pine beetle outbreaks and drought on severe wildfires in northwestern Colorado and southern Wyoming: A look at the past century. *Forest Ecology and Management*, *262*(9), 1686-1696. https://doi.org/10.1016/j.foreco.2011.07.016

Lynch, H. J., Renkin, R. A., Crabtree, R. L., & Moorcroft, P. R. (2006). The influence of previous mountain pine beetle (Dendroctonus ponderosae) activity on the 1988 Yellowstone fires. *Ecosystems*, *9*(8), 1318-1327.

McCarley, T. R., Kolden, C. A., Vaillant, N. M., Hudak, A. T., Smith, A. M., & Kreitler, J. (2017). Landscape-scale quantification of fire-induced change in canopy cover following mountain pine beetle outbreak and timber harvest. *Forest Ecology and Management*, *391*, 164-175.

Meigs, G. W., Campbell, J. L., Zald, H. S., Bailey, J. D., Shaw, D. C., & Kennedy, R. E. (2015). Does wildfire likelihood increase following insect outbreaks in conifer forests? *Ecosphere*, *6*(7), 1-24.

Meigs, G. W., Zald, H. S., Campbell, J. L., Keeton, W. S., & Kennedy, R. E. (2016). Do insect outbreaks reduce the severity of subsequent forest fires? *Environmental Research Letters*, *11*(4), 045008.

Mietkiewicz, N., & Kulakowski, D. (2016). Relative importance of climate and mountain pine beetle outbreaks on the occurrence of large wildfires in the western USA. *Ecological Applications*, *26*(8), 2525-2537.

Millar, C. I., & Delany, D. L. (2019). Interaction between mountain pine beetle-caused tree mortality and fire behavior in subalpine whitebark pine forests, eastern Sierra Nevada, CA; Retrospective observations. *Forest Ecology and Management*, *447*, 195-202.

Moriarty, K., Cheng, A. S., Hoffman, C. M., Cottrell, S. P., & Alexander, M. E. (2019). Firefighter observations of "surprising" fire behavior in mountain pine beetle-attacked lodgepole pine forests. *Fire*, *2*(2), 34.

Nelson, M. F., Ciochina, M., & Bone, C. (2016). Assessing spatiotemporal relationships between wildfire and mountain pine beetle disturbances across multiple time lags. *Ecosphere*, *7*(10), e01482.

Page, W., & Jenkins, M. J. (2007). Predicted fire behavior in selected mountain pine beetle–infested lodgepole pine. *Forest Science*, *53*(6), 662-674.

Page, W. G., Jenkins, M. J., & Runyon, J. B. (2012). Mountain pine beetle attack alters the chemistry and flammability of lodgepole pine foliage. *Canadian Journal of Forest Research*, *42*(8), 1631-1647.

Perrakis, D. D., Lanoville, R. A., Taylor, S. W., & Hicks, D. (2014). Modeling wildfire spread in mountain pine beetle-affected forest stands, British Columbia, Canada. *Fire Ecology*, *10*(2), 10-35.

Schoennagel, T., Veblen, T. T., Negron, J. F., & Smith, J. M. (2012). Effects of mountain pine beetle on fuels and expected fire behavior in lodgepole pine forests, Colorado, USA. *PLoS One*, *7*(1), e30002.

Sieg, C. H., Linn, R. R., Pimont, F., Hoffman, C. M., McMillin, J. D., Winterkamp, J., & Baggett, L. S. (2017). Fires following bark beetles: Factors controlling severity and disturbance interactions in ponderosa pine. *Fire Ecology*, *13*(3), 1-23.

Simard, M., Romme, W. H., Griffin, J. M., & Turner, M. G. (2011). Do mountain pine beetle outbreaks change the probability of active crown fire in lodgepole pine forests? *Ecological Monographs*, *81*(1), 3-24.

Talucci, A. C., & Krawchuk, M. A. (2019). Dead forests burning: the influence of beetle outbreaks on fire severity and legacy structure in sub-boreal forests. *Ecosphere*, *10*(5), e02744.
